# First-principles prediction of the electronic properties and contact features of graphene/γ-GeSe van der Waals heterostructure: effects of electric fields and strains

**DOI:** 10.1039/d4ra06977c

**Published:** 2024-11-28

**Authors:** Tuan V. Vu, A. I. Kartamyshev, A. A. Lavrentyev, Nguyen N. Hieu, Huynh V. Phuc, Chuong V. Nguyen

**Affiliations:** a Laboratory for Computational Physics, Institute for Computational Science and Artificial Intelligence, Van Lang University Ho Chi Minh City Vietnam tuan.vu@vlu.edu.vn; b Faculty of Mechanical – Electrical and Computer Engineering, School of Technology, Van Lang University Ho Chi Minh City Vietnam; c Department of Electrical Engineering and Electronics, Don State Technical University 1 Gagarin Square 344010 Rostov-on-Don Russian Federation; d Institute of Research and Development, Duy Tan University Da Nang 550000 Vietnam hieunn@duytan.edu.vn; e Faculty of Natural Sciences, Duy Tan University Da Nang 550000 Vietnam; f Division of Physics, School of Education, Dong Thap University Cao Lanh 870000 Vietnam hvphuc@dthu.edu.vn; g Department of Materials Science and Engineering, Le Quy Don Technical University Hanoi 100000 Vietnam; h Center for Mechanism and Material Science, Le Quy Don Technical University Hanoi 100000 Vietnam

## Abstract

In this work, we investigate systematically the electronic properties and tunable contact behavior of the graphene/γ-GeSe heterostructure under applied electric fields and out-of-plane strains using first-principles calculations. At equilibrium, the heterostructure forms a p-type Schottky contact with low Schottky barrier, making it suitable for low-resistance electronic devices. The application of electric fields modulates the Schottky barriers, enabling transitions between p-type and n-type contacts and even Schottky to Ohmic contact. Similarly, strain engineering by adjusting the interlayer spacing effectively alters the contact types, with compressive strain reducing the Schottky barrier to zero, and tensile strain inducing a shift from p-type to n-type Schottky contact. Our findings provide a pathway for optimizing graphene/γ-GeSe heterostructures for multifunctional applications, emphasizing tunable electronic properties to enhance device performance.

## Abstract 2

In this work, we investigate systematically the electronic properties and tunable contact behavior of the graphene/γ-GeSe heterostructure under applied electric fields and out-of-plane strains using first-principles calculations.

## Graphical abstract

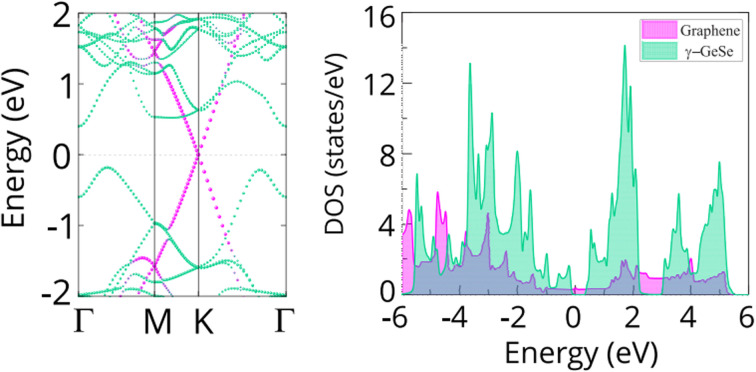

## 1 Introduction

The advent of two-dimensional (2D) materials has led the way for a new era of electronic and optoelectronic devices, due to their remarkable physical properties.^1–3^ Among the diverse array of 2D materials, graphene stands out for its exceptional electronic properties and carrier transport.^4^ However, while graphene's superior electronic properties are well-established, its lack of an intrinsic band gap hinders its application in digital electronics, particularly in logic devices and transistors that require precise on-off switching characteristics.^5^ To address this limitation, the exploration of novel 2D materials with sizable band gaps has emerged as a promising strategy. Novel 2D materials such as transition metal mono- and di-chalcogenides^6–9^ offer tunable bandgaps, making them ideal candidates for applications ranging from photodetectors to field-effect transistors.

Currently, the integration of these novel 2D materials into device architectures presents both opportunities and challenges. A crucial aspect of optimizing device performance involves the development of efficient metal–semiconductor (M–S) heterostructures that facilitate effective charge transport while minimizing contact resistance. The nature of the contact – whether Ohmic or Schottky – significantly impacts charge injection efficiency and overall device performance. Recent advances in fabricating and characterizing 2D/2D M–S heterostructures underscore their potential to achieve low contact resistance and enhanced functionality, thereby advancing the capabilities of 2D material-based technologies. Recently, researchers have focused on exploring novel configurations and materials to push the boundaries of device performance. The integration of electrical graphene contact with 2D semiconductor channels, such as GaSe,^10,11^ MoS_2_,^12,13^ borophene,^14^ GeC^15^ and MoSi_2_N_4_ (ref. 16 and 17) has shown promising results. Although graphene is a gapless semiconductor, its exceptional electrical conductivity demonstrates that employing graphene as an electrode in M–S heterostructures significantly enhances device performance. The integration of graphene with various 2D semiconductors not only optimizes the electronic properties of the heterostructure but also opens new avenues for developing high-performance electronic and optoelectronic devices.

Furthermore, recent advancements have expanded the landscape of 2D materials with the introduction of Janus materials, significantly broadening the potential applications of 2D materials.^18,19^ Janus materials, distinguished by their asymmetrical structure featuring different atomic species on each side, offer unique electronic and optical properties due to this inherent asymmetry. Among those, group-IV monochalcogenides like γ-GeSe have been successfully synthesized *via* chemical vapor deposition (CVD),^20^ presenting new opportunities for applications requiring high carrier mobility and adjustable electronic characteristics. Jang *et al.*^21^ demonstrated that γ-GeSe, a unique polymorph, possess high electrical conductivity coupled with a low Seebeck coefficient. Zhang *et al.*^22^ suggested that the electronic properties and conductivity of γ-GeSe are adjustable upon the application of strain engineering. All these findings suggest that γ-GeSe can act as a promising semiconducting channel for the integration with graphene electrode in the M–S heterostructure.

Herein, we designed graphene/γ-GeSe heterostructure and considered its characteristics under external electric fields and vertical strains. The graphene/γ-GeSe the heterostructure exhibits tunable contact barriers and types. These findings highlight the versatility of graphene/γ-GeSe heterostructure, making it a promising material for advanced devices with adjustable performance characteristics.

## 2 Computational methods

The first-principles calculations in this study were performed using density functional theory (DFT) using the Quantum Espresso package.^23,24^ The exchange-correlation effects were treated using the generalized gradient approximation (GGA)^25^ with the Perdew–Burke–Ernzerhof (PBE) functional.^26^ To describe the interactions between electrons and ions, the projector augmented-wave (PAW) method^27,28^ was employed. An energy cutoff of 510 eV was chosen to ensure accurate convergence of the total energies. Additionally, a density cutoff, set at four times the energy cutoff, was applied during the calculations. A vacuum layer of 30 Å was applied to prevent interactions between periodic images. The geometry was fully optimized until the forces and total energies acting on each atom were smaller than 0.01 eV Å^−1^ and 10^−6^ eV, respectively. For sampling the reciprocal space, we used a 9 × 9 × 1 Monkhorst–Pack *k*-point grid, ensuring precision in both the structural optimizations and electronic structure calculations. To account for van der Waals (vdW) forces between the graphene and γ-GeSe layers, we applied the DFT-D3 correction,^29^ using the damping function parameters of *s*_8_ = 0.72 and *s*_R,6_ = 1.22, to accurately capture the interlayer interactions. It should be noted that the DFT-D3 method was chosen for its balance of accuracy and computational efficiency. Moreover, the DFT-D3 method has been widely adopted and validated in the previous reports,^30–34^ making it a reliable choice for our heterostructure. To obtain a more accurate band gap for the 2D semiconductor, the Heyd–Scuseria–Ernzerhof (HSE06) method^35^ was employed, utilizing a specific mixing parameter of 0.25. The *ab initio* molecular dynamics (AIMD) simulations were carried out using a canonical ensemble (NVT) along with the Nosé–Hoover thermostat.^36^ We utilized 3 × 3 × 1 supercells of graphene/γ-GeSe heterostructure, conducting the simulations at room temperature of 300 K over 5 ps with a time step of 1 fs. The phonon calculations were performed using the Phonopy package^37,38^ in the framework of the density-functional-perturbation theory (DFPT) method^39^ using 4 × 4 × 1 supercell of materials.

## 3 Results and discussion

**Fig. 1:**
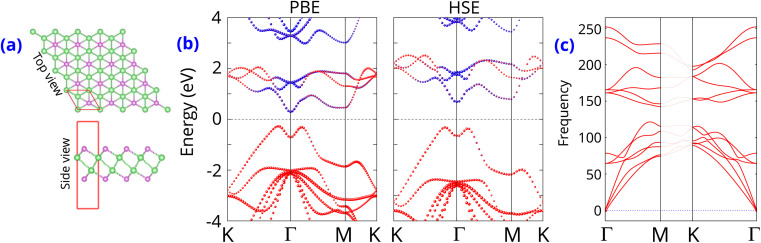
(a) Atomic structure (b) band structures and (c) phonon spectra of γ-GeSe monolayer.

First, the intrinsic properties of the perfect γ-GeSe monolayer are examined initially. The atomic structure of γ-GeSe monolayer belongs to the *F*3̄*m*1space group of crystal symmetry, as shown in Fig. 1(a). Each unit cell of γ-GeSe monolayer consists of four different sublayers arranged as Se–Ge–Ge–Se. The calculated lattice constant of γ-GeSe is obtained to be 3.78 Å, which is good agreement with the experiment and computational values.^20,40^ The band structures calculated by PBE and HSE method is displayed in Fig. 1(b). The γ-GeSe presents a semiconductor charaterized by an indirect band gap, according to both HSE and PBE functional predictions. The lowest conduction band is found at the Γ point, while the highest valence band lies along the K–Γ path. The band gap of γ-GeSe monolayer is 0.57 (1.0) eV using the PBE (HSE) functional. γ-GeSe monolayer is predicted to be dynamically stable because all its frequencies are positive in the whole phonon dispersion spectrum, as depicted in Fig. 1(c). In addition, the phonon spectrum reveals a total of 12 branches, including 3 acoustic branches.

**Fig. 2:**
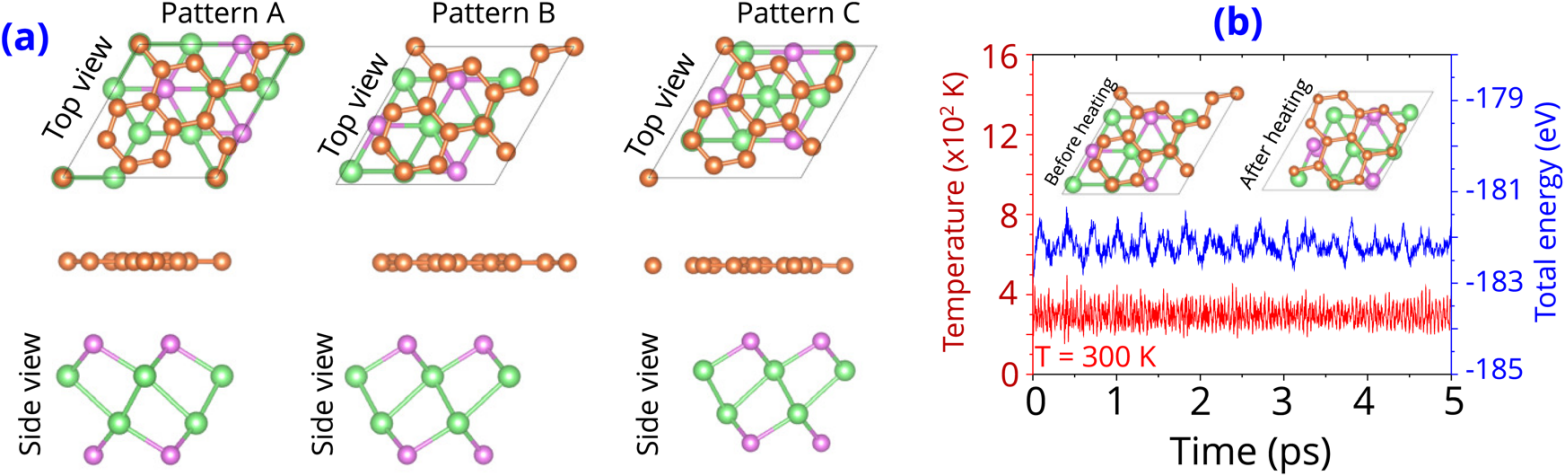
(a) Atomic structures of the graphene/γ-GeSe heterostructure for different patterns. (b) AIMD simulation of the fluctuations in the total energy and temperature of the graphene/γ-GeSe heterostructure. The insets show the geometric structure the graphene/γ-GeSe heterostructure before and after the relaxation times up to 5 ps.

The atomic structure of combined graphene/γ-GeSe heterostructure is illustrated in Fig. 2(a). The γ-GeSe heterostructure consists of 3 × 3 × 1 graphene unit cells and 2 × 2 × 1 γ-GeSe unit cells. The graphene/GeSe heterostructure was constructed by simply stacking the graphene and GeSe layers along the vertical direction, without rotating the graphene layer. After stacking, we then shifted the graphene layer laterally over the GeSe layer to produce the patterns A, B, and C, which are illustrated in Fig. 2(a). In constructing the heterostructure, the lattice parameter of the GeSe layer is fixed, while the lattice parameter of the graphene layer is adjusted through stretching. Following construction, the heterostructure undergoes full relaxation, allowing the unit cell shape, volume, and ions to relax. The resulting lattice parameter of the heterostructure is 7.48 Å, indicating a minor lattice mismatch of approximately 1.5%. The interlayer spacing d is obtained to be 3.49 Å for pattern A, 3.50 Å for pattern B and 3.50 Å for pattern C. These values are in the same magnitude as those in other graphene heterostructures.^31,41,42^ These obtained *d* values are comparable to those observed in other graphene-based heterostructures.^31,41,42^ This observation suggests that the graphene/γ-GeSe heterostructure falls within the of vdW systems, where the two layers interact through weak vdW forces. Furthermore, we calculated the binding energy *E*_b_ to access the stability of the graphene/γ-GeSe heterostructure as follows:1
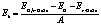
Here, the total energy *E*_G/γ-GeSe_ stands for the graphene/γ-GeSe heterostructure, *E*_G_ and *E*_γ-GeSe_ stand for the graphene and γ-GeSe. The symbol *A* refers to the surface area of the heterostructure. The obtained *E*_b_ are −50.1, −51.2 and −48.6 meV Å^−2^, respectively, for the pattern A, B and C. The negative value of the *E*_b_ specifies the stability of the heterostructure for all patterns. The *E*_b_ for the pattern B is the lowest, signifying that this pattern is the most energetically favorable. Furthermore, the AIMD simulation is also performed to access the thermal stability of the most energetically favorable pattern B. The fluctuations in total energy and temperature of the graphene/γ-GeSe heterostructure are shown in Fig. 2(b), demonstrating minimal variations in both parameters. In addition, the atomic structure of the graphene/γ-GeSe heterostructure under the relaxation time of 5 ps is well sustained and shows no significant distortions. This finding indicates that the graphene/γ-GeSe heterostructure exhibits thermal stability at room temperature.

**Fig. 3:**
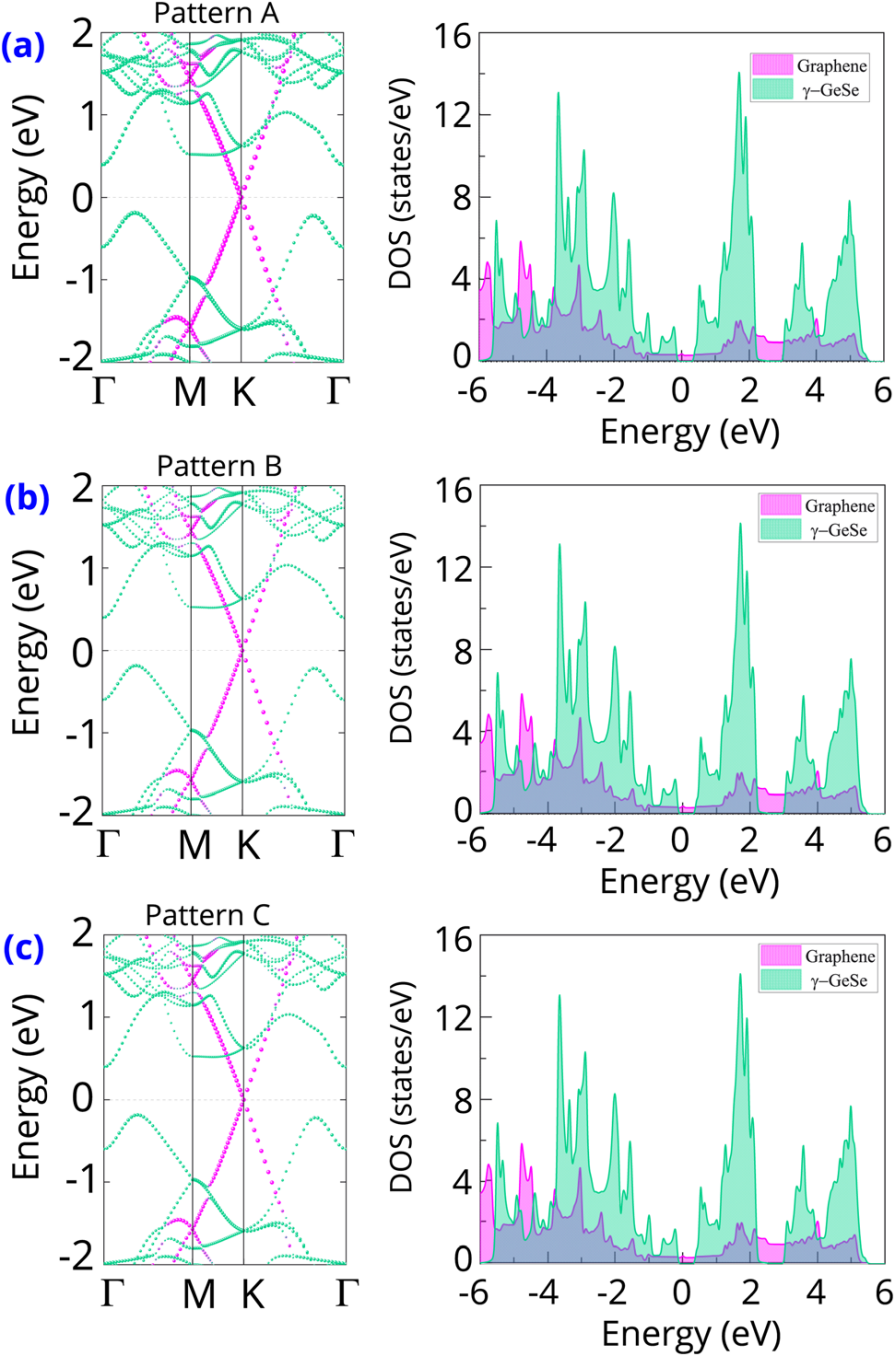
The weighted projections in the band structures and density of states for each layer of the graphene/γ-GeSe heterostructure for the different patterns of (a) pattern A, (b) pattern B and (c) pattern C. Green and purple balls denote the weighted projections of the γ-GeSe and graphene layers, respectively.

The band structure projections of the graphene/γ-GeSe heterostructure are presented in Fig. 3, highlighting several interesting phenomena. Firstly, the Dirac cone of the graphene layer remains at the K point, indicating that graphene retains the intrinsic electronic properties even when combined with the γ-GeSe layer. This preservation of intrinsic characteristics in graphene is crucial for its functionality in various electronic applications. Secondly, there is a noticeable shift in the valence band maximum of the γ-GeSe layer from the K–Γ path to the Γ–M path. This shift is attributed to the effect of the band folding that arises due to the supercell size in the heterostructure. Similar effects have been noted in other vdW heterostructures.^43–45^ Thirdly, the band structures of patterns A, B, and C are very similar. This observation suggests that the electronic properties of the graphene/γ-GeSe heterostructure are not significantly influenced by stacking configurations. Finally, the positioning of the Fermi level (*E*_F_) between the conduction and valence bands of the γ-GeSe layer indicates the formation of a Schottky contact with the p-type and n-type Schottky barriers:

2 *Φ*_p_ = *E*_F_ − *E*_VBM_

and

3 *Φ*_n_ = *E*_CBM_ − *E*_F_

The band edges of the γ-GeSe layer are highlighted by the *E*_VBM_ and *E*_CBM_, respectively. The *E*_g_ ≈ *Φ*_p_ + *Φ*_n_ is the γ-GeSe band gap in the heterostructure. The *E*_VBM_ is closer to the *E*_F_ than the *E*_CBM_, which clearly indicates the formation of p-type Schottky contact. The calculated Schottky barriers *Φ*_n_ and *Φ*_p_ are 0.40 eV and 0.19 eV, respectively. The relatively lower *Φ*_p_ compared to *Φ*_n_ confirms the p-type nature of the contact. Importantly, the low *Φ*_p_ barrier suggests that the graphene/γ-GeSe heterostructure can serve as an efficient electrode with minimal contact resistance. Additionally, the Schottky barrier in this heterostructure is significantly lower than those reported in other heterostructures, such as graphene/BiI_3_ (0.53 eV),^31^ graphene/GeTe (0.68 eV),^41^ and graphene/HfN_2_ (0.67 eV).^46^ This further highlights the advantages of the graphene/γ-GeSe heterostructure for low-resistance electronic applications. Furthermore, the density of states (DOS) of the graphene and γ-GeSe layers in the graphene/γ-GeSe heterostructure are also calculated and plotted in Fig. 3. The orbital states of the γ-GeSe layer are absent in the Fermi state of the graphene/γ-GeSe heterostructure, indicating the weak Fermi level pinning.

Furthermore, the charge transfers and charge redistribution always occur at the interface of the graphene/γ-GeSe heterostructure. The mechanism of the charge redistribution can be visualized by analyzing the differencial charge densities (CDD) between the graphene/γ-GeSe (*ρ*_G/γ-GeSe_) and the isolated graphene *ρ*_G_ and γ-GeSe (*ρ*_γ-GeSe_) monolayers as follows:

4 Δ*ρ* = *ρ*_G/γ-GeSe_ − *ρ*_G_ − *ρ*_γ-GeSe_

**Fig. 4:**
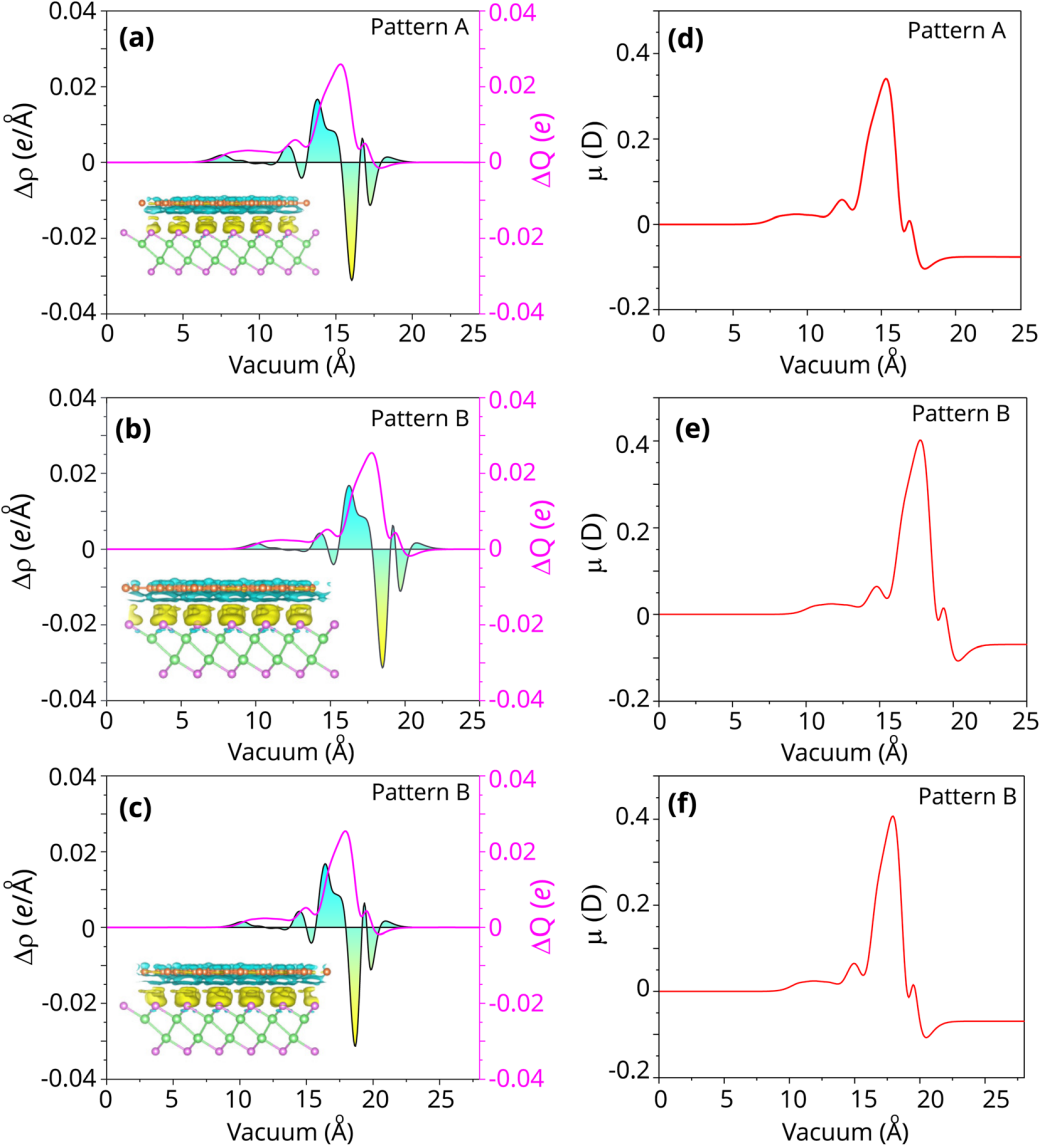
Planar-average charge density difference and total amount of charge transfers of (a) pattern A, (b) pattern B and (c) pattern C. Interfacial dipole moment *μ* of (d) pattern A, (e) pattern B and (f) pattern C of the graphene/γ-GeSe heterostructure. The charge accumulation (depletion) is denoted by the yellow (cyan) area.

The CDD in the graphene/-GeSe heterostructure for all patterns are visualized in Fig. 4. The positive values of the charge transfers represent the charge accumulation, while the negative values show the charge depletion. By analyzing the planar 2D and 3D CDD of the graphene/γ-GeSe heterostructure for all patterns, we find that the negative values of charges are occurred in the graphene layer, while the positive values are obtained in the γ-GeSe layer. The charge accumulation is observed in the γ-GeSe layer, while the charge depletion is occurred in the graphene layer. This means that the electrons are transferred from the graphene to the γ-GeSe layer in the graphene/γ-GeSe heterostructure for all stacking patterns. The transferred electrons can be obtained as:5
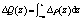


The transferred electrons is about 0.025*e*. One should note that the charge transfers includes an built-in interfacial dipole. The interfacial dipole moment can be calculated and described as follows:6
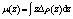


The interfacial dipole moment for the graphene/γ-GeSe heterostructure is obtained to be −0.1 debye, which confirms the formation of the interface dipole of the heterostructure. Additionally, a negative value of the interfacial dipole moment specifies that the graphene layer losses electrons, while the γ-GeSe layer gains them.

**Fig. 5:**
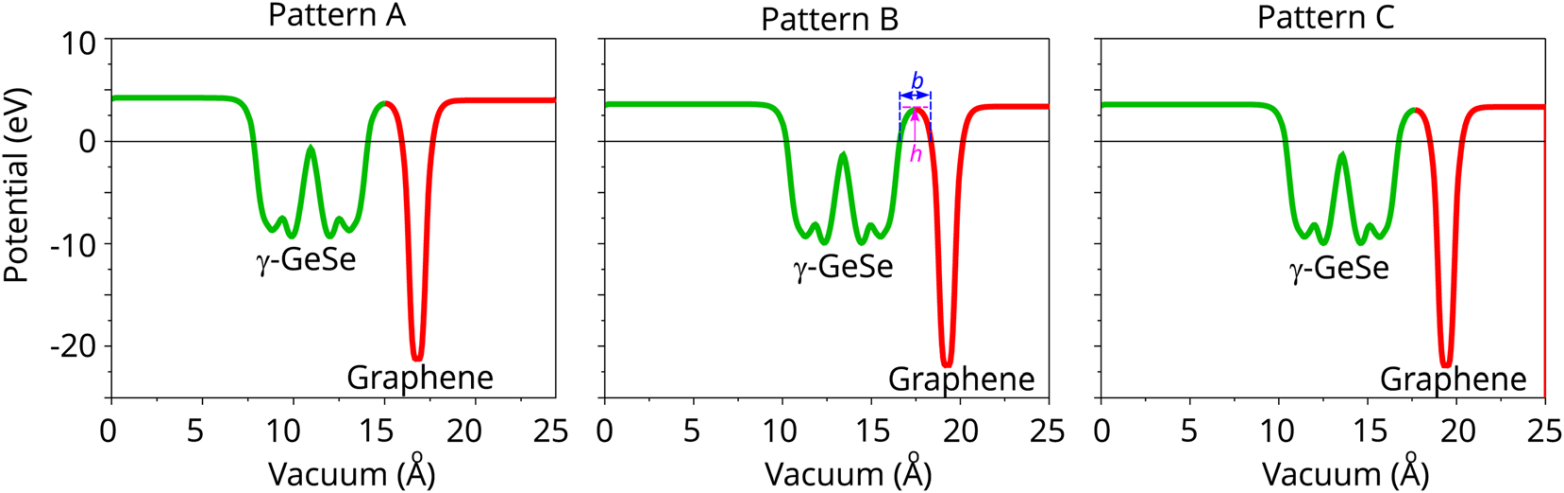
Electrostatic potential of the graphene/γ-GeSe heterostructure for the different patterns.

Furthermore, the planar-averaged electrostatic potentials for all stacking patterns are calculated and depicted in Fig. 5. The transfer of charge across the graphene/γ-GeSe heterostructure results in the formation of an interfacial dipole. This dipole can be determined by the difference in work functions between the heterostructure *ϕ*_H_ and graphene *ϕ*_G_, as follows:

7 Δ*V* = *ϕ*_H_ − *ϕ*_G_

The calculated interface dipole for the graphene/γ-GeSe heterostructure is obtained to be 0.24 eV for all the stacking patterns. The lower work function of graphene compared to the γ-GeSe layer confirms that the electrons flow from the graphene to the γ-GeSe layer. The small value of the interface dipole of 0.24 eV in the graphene/γ-GeSe heterostructure suggests that the Fermi level is depinned, allowing for more efficient charge carrier across the heterostructure. More interestingly, analyzing the potential allows us to determine the tunneling height (*h*) and width (*b*) of the heterostructure, as shown in Fig. 5. The calculated tunneling barriers *h*/*b* of the graphene/γ-GeSe heterostructure for the pattern A, B and C are obtained to be 3.656/1.83, 3.046/1.76 and 3.013/1.75 eV Å^−1^, respectively. Remarkably, reducing the barrier height (*h*) and barrier width (*b*) in the metal–semiconductor heterostructure enhances electron injection efficiency, thereby improving device performance. Consequently, we calculate the tunneling probability in the heterostructure as follows:8
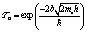
Here, h is the reduced Planck constant, *m*_e_ is the mass of free electron. The calculated 
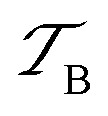
 of the graphene/γ-GeSe heterostructure for the pattern A, B and C are estimated to be 2.7, 4.2 and 4.4%, respectively. These low values of 
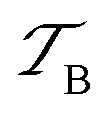
indicate that the heterostructure shows low electron transparency. Based on the 
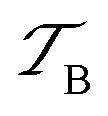
, the tunneling-specific contact resistivity *ρ*_*t*_ can be obtained as follows:9
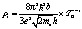


The charge injection efficiency in the heterostructure is influenced by the obtained *ρ*_*t*_ values, which are 7.3 × 10^−10^, 4.9 × 10^−10^ and 4.7 × 10^−10^ Ω cm^−2^, respectively. These low *ρ*_*t*_ values are comparable to those obtained in 3D metals/2D semiconductors,^47^ highlighting the potential of this heterostructure as an efficient semimetal contact material.

**Fig. 6:**
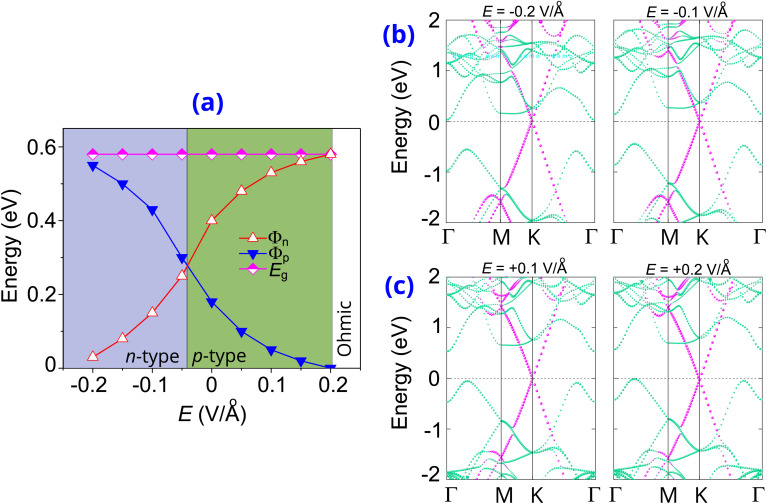
(a) The evolution of the Schottky barriers in the graphene/γ-GeSe heterostructure under different values of electric fields. The projections of weighted bands of the graphene/γ-GeSe heterostructure under the application of (b) negative and (c) positive values of electric field. Green and purple lines denote the projections of the γ-GeSe and graphene layers, respectively.

Furthermore, we focus on the effects of electric fields *E* and vertical strain on the intrinsic properties of the graphene/γ-GeSe heterostructure. The positive *E*-direction is oriented from the γ-GeSe toward the graphene layer with a field strength ranging from −0.2 to +0.2 V Å^−1^. The evolution of the Schottky barriers in the graphene/γ-GeSe heterostructure is illustrated in Fig. 6(a), which specifies the changes in the Schottky barriers of the graphene/γ-GeSe heterostructure in two distinct directions. A negative electric field decreases the *Φ*_n_ Schottky barrier while increasing the *Φ*_p_ Schottky barrier. Conversely, a positive electric field increases *Φ*_n_ and reduces *Φ*_p_. This finding highlights the versatility of the Schottky barriers in the graphene/γ-GeSe heterostructure, which can be effectively adjusted and even reduced to zero, specifying the conversion from Schottky contact to Ohmic contact. From Fig. 6, the *Φ*_p_ Schottky barrier is reached zero under *E* = +0.2 V Å^−1^, while the *Φ*_n_ barrier can approach zero when a negative *E* is below −0.2 V Å^−1^. Specifically, the *Φ*_n_ barrier reaches zero at *E* = −0.24 V Å^−1^. Consequently, applying electric fields can induce the Schottky–Ohmic conversion in the graphene/γ-GeSe heterostructure. Furthermore, the evolution in the Schottky barriers in the graphene/γ-GeSe heterostructure also indicates that the *Φ*_n_ Schottky barrier narrows and becomes smaller than the *Φ*_p_, enabling a transition in the contact types. From Fig. 6(a), the *Φ*_n_ of the graphene/γ-GeSe heterostructure reduces and becomes smaller than the *Φ*_p_ under *E* = −0.05 V Å^−1^, enabling a p-type to n-type transition. All these findings specify that the electric fields induces the evolution in the contact barriers and drive in the contact types transition. These findings make the graphene/γ-GeSe heterostructure. This tunability highlights the potential applications of the graphene/γ-GeSe heterostructure for versatile electronic Schottky devices.

To elucidate the physical mechanisms driving the evolution in the Schottky barriers of the heterostructure, we examine the weighted band projections under varying electric field strengths, which presented in Fig. 6(b and c). The results reveal that a negative *E* drives the band edges of the γ-GeSe layer from the higher to the lower energy. Specifically, when the negative *E* is reduced below −0.05 V Å^−1^, the CBM of the γ-GeSe shifts closer to the Fermi level than the VBM, marking a transition from p-type to n-type contact. Further decreasing the electric field to below −0.2 V Å^−1^ results in the CBM at the Γ point crossing the Fermi level, indicating a transition from Schottky to Ohmic contact. Additionally, the weighted band projections under a positive electric field are depicted in Fig. 6(c), revealing notable changes in the γ-GeSe band edges. Applying a positive electric field shifts the γ-GeSe band edges to higher energy: with increasing field strength, the CBM shifts further from the Fermi level, while the VBM shifts less significantly. This causes a widening of the *Φ*_n_ barrier and a reduction in the *Φ*_p_ barrier. At a positive field of +0.2 V Å^−1^, this shift can drive the VBM across the Fermi level, enabling a transition to Ohmic from its Schottky.

**Fig. 7:**
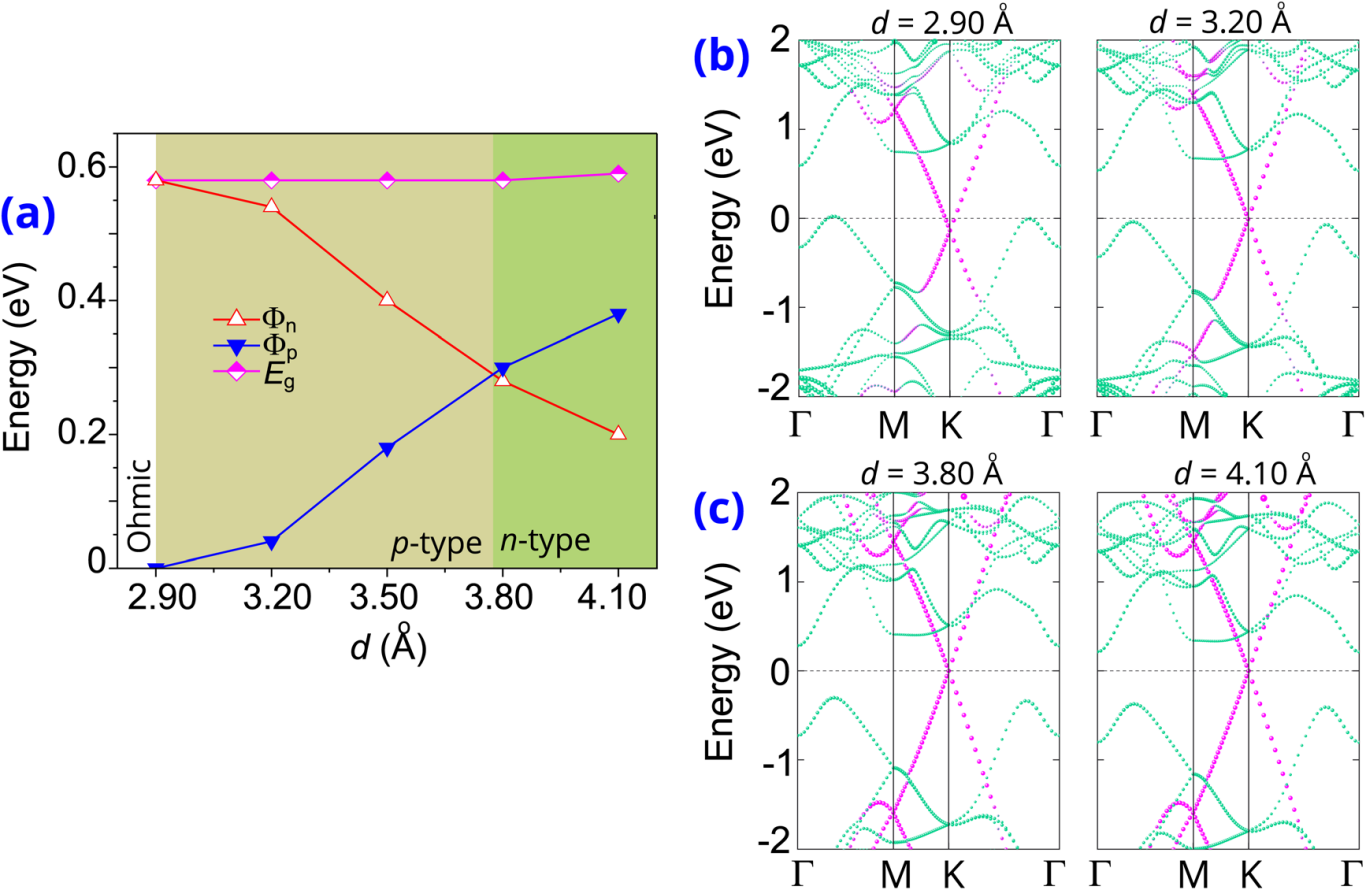
(a) The evolution of the Schottky barriers in the graphene/γ-GeSe heterostructure under different interlayer spacings *d*. The projections of weighted bands of the graphene/γ-GeSe heterostructure with (b) decreasing and (c) increasing the interlayer spacings. Green and purple lines denote the projections of the γ-GeSe and graphene layers, respectively.

The effects of the out-of-plane strain are considered by adjusting the interlayer spacings *d*. Compressive strain is defined by a reduction in interlayer spacing, whereas tensile strain is indicated by an increase in this spacing. Similar to the effects of the electric field, this strain also enables the versatile contact barriers and types. The evolution of the Schottky barriers of the graphene/γ-GeSe heterostructure is displayed in Fig. 7(a). Reducing the interlayer spacing from the equilibrium value of *d* = 3.50 Å down to 2.90 Å facilitates a transition from Schottky contact to Ohmic contact by effectively reducing the *Φ*_p_ Schottky barrier to zero. This signifies that the compressive strain causes to the reduced in the *Φ*_p_ barrier, while the *Φ*_n_ barrier increases. In contrast, tensile strain, achieved by increasing the interlayer spacing (*d*), raises the *Φ*_p_ barrier and lowers the *Φ*_n_ barrier. At the *d* = 3.80 Å, the Schottky barrier *Φ*_p_ becomes larger than the *Φ*_n_ of the graphene/γ-GeSe heterostructure, signifying a p- to n-type contact's transition.

The projections of weighted bands of the graphene/γ-GeSe heterostructure under different *d* are illustrated in Fig. 7(b and c) for better understanding the physical mechanism driving changes in Schottky barriers and contact types. The band projections of the graphene/γ-GeSe heterostructure illustrate how out-of-plane strain alters the band edges of the γ-GeSe layer in the graphene/γ-GeSe heterostructure. By reducing *d*, the band edges of the γ-GeSe layer are up-shifted, resulting in a lower *Φ*_p_ and a higher *Φ*_p_ barrier. At *d* = 2.90 Å, the VBM band edge of the γ-GeSe layer moves upward and crosses the Fermi level, specifying a Schottky to Ohmic conversion. Moreover, by increasing *d*, the band edges of the γ-GeSe layer turn to shift downward, resulting in a lower *Φ*_n_ and a higher *Φ*_p_ Schottky barrier. At *d* = 3.80 Å, the γ-GeSe CBM aligns closer to the Fermi level than the VBM, enabling a transition from p-type to n-type contact. Notably, the transitions between different Schottky and Ohmic contacts, as well as p to n-type contact, are crucial for tailoring the electronic properties and contact characteristics of the graphene/γ-GeSe heterostructure. These transitions enable precise control over charge carrier transport at the interface, which is vital for optimizing device performance in electronic and optoelectronic applications. The ability to induce such transitions through external electric fields and strain offers a versatile platform for designing tunable contacts, improving efficiency, and enabling multifunctional device capabilities in next-generation applications.

## 4 Conclusions

In conclusion, we have explored the electronic properties and tunable contact behavior of the graphene/γ-GeSe heterostructure using first-principles calculations. The graphene/γ-GeSe heterostructure forms a p-type Schottky contact with a low barrier of 0.19 eV, making it highly suitable for low-resistance electronic applications. Furthermore, the application of electric fields allows for precise modulation of the Schottky barriers, enabling transitions between p-type and n-type Schottky contacts, and even transforming Schottky contact into Ohmic one. Strain engineering, achieved by adjusting the interlayer spacing, provides additional control over the contact behavior, facilitating similar transitions. These findings underscore the versatility of the graphene/γ-GeSe heterostructure, making it a promising candidate for the design of next-generation electronic devices with enhanced performance.

## Data availability

The data that support the findings of this study are available from the corresponding author upon reasonable request.

## Conflicts of interest

There are no conflicts to declare.
